# Chemical Comparison and Identification of Xanthine Oxidase Inhibitors of Dioscoreae Hypoglaucae Rhizoma and Dioscoreae Spongiosae Rhizoma by Chemometric Analysis and Spectrum–Effect Relationship

**DOI:** 10.3390/molecules28248116

**Published:** 2023-12-15

**Authors:** Guo Rui, Zhang-Yi Qin, Ya-Qing Chang, Yu-Guang Zheng, Dan Zhang, Li-Min Yao, Long Guo

**Affiliations:** 1 Traditional Chinese Medicine Processing Technology Innovation Center of Hebei Province, Hebei University of Chinese Medicine, Shijiazhuang 050200, China; guo_123rui@163.com (G.R.); helios20001005@163.com (Z.-Y.Q.); czuoya_123@163.com (Y.-Q.C.); zyg314@163.com (Y.-G.Z.); zhangdan@hebcm.edu.cn (D.Z.); 2 International Joint Research Center on Resource Utilization and Quality Evaluation of Traditional Chinese Medicine of Hebei Province, Hebei University of Chinese Medicine, Shijiazhuang 050200, China; 3 Department of Pharmaceutical Engineering, Hebei Chemical & Pharmaceutical College, Shijiazhuang 050026, China; 4 Bethune International Peace Hospital, Shijiazhuang 050082, China

**Keywords:** Dioscoreae hypoglaucae Rhizoma, Dioscoreae spongiosae Rhizoma, xanthine oxidase, chemometric analysis, spectrum–effect relationship

## Abstract

Dioscoreae hypoglaucae Rhizoma (DH) and Dioscoreae spongiosae Rhizoma (DS) are two similar Chinese herbal medicines derived from the Dioscorea family. DH and DS have been used as medicines in China and other Asian countries for a long time, but study on their phytochemicals and bioactive composition is limited. This present study aimed to compare the chemical compositions of DH and DS, and explore the anti-xanthine oxidase components based on chemometric analysis and spectrum–effect relationship. Firstly, an HPLC method was used to establish the chemical fingerprints of DH and DS samples, and nine common peaks were selected. Then, hierarchical clustering analysis, principal component analysis and orthogonal partial least squares discriminant analysis were employed to compare and discriminate DH and DS samples based on the fingerprints data, and four steroidal saponins compounds (protodioscin, protogracillin, dioscin, gracillin) could be chemical markers responsible for the differences between DH and DS. Meanwhile, the anti-xanthine oxidase activities of these two herbal medicines were evaluated by xanthine oxidase inhibitory assay in vitro. Pearson correlation analysis and partial least squares regression analysis were subsequently used to investigate the spectrum–effect relationship between chemical fingerprints and xanthine oxidase inhibitory activities. The results showed that four steroidal saponins, including protodioscin, protogracillin, methyl protodioscin and pseudoprogracillin could be potential anti-xanthine oxidase compounds in DH and DS. Furthermore, the xanthine oxidase inhibitory activities of the four selected inhibitors were validated by anti-xanthine oxidase inhibitory assessment and molecular docking experiments. The present work provided evidence for understanding of the chemical differences and the discovery of the anti-xanthine oxidase constituent of DH and DS, which could be useful for quality evaluation and bioactive components screening of these two herbal medicines.

## 1. Introduction

Hyperuricemia is a common metabolic disease characterized by an elevated level of uric acid, which has been recognized as a risk factor for inducing many other diseases, such as gout, hyperlipidemia, hypertension, diabetes, chronic renal dysfunction and cardiovascular disorders [1,2,3]. Hyperuricemia is mainly caused by underexcretion and/or overproduction of uric acid. Xanthine oxidase is a key enzyme involved in uric acid generation and purine metabolism, which can catalyze the oxidation of xanthine or hypoxanthine to uric acid and participate in the production and oxidation of uric acid [4,5]. Currently, the anti-hyperuricemic drugs are mainly divided into two types, including xanthine oxidase inhibitors (allopurinol, febuxostat, topiroxostat) and uricosuric agents (benzbromarone) [6,7]. However, long-term use of these drugs could cause various adverse effects, such as allergic dermatitis, liver function damage, gastrointestinal reactions, blood abnormalities and lymphadenopathy [8,9,10]. Therefore, development of new and safe xanthine oxidase inhibitors is urgently needed for treatment of hyperuricemia.

Recently, several herbal medicines, such as Phellodendri amurensis Cortex, Atractylodis Rhizoma and Dioscoreae nipponicae Rhizoma, which are multi-target and low toxicity, have been proven to have good therapeutic effects on hyperuricemia [11]. Some chemical constituents isolated from herbal medicines, including alkaloids, phenolics and saponins are also shown to have advantages and good prospects in the prevention and treatment of hyperuricemia [12]. Thus, there has been a great interest in the discovery of xanthine oxidase inhibitors from herbal medicines. Dioscoreae hypoglaucae Rhizoma (DH) and Dioscoreae spongiosae Rhizoma (DS) are two similar Chinese herbal medicines derived from the Dioscorea family. These two herbal medicines have the effect of dampness and turbidity, dispelling wind and removing paralysis, and have been widely applied in the treatment of rheumatism, joint pain, hyperuricemia and cardiovascular diseases [13]. Modern phytochemical and pharmacological studies showed that steroidal saponins are the main bioactive constituents both in DH and DS, and the steroidal saponins contained in these two herbal medicines showed multiple biological activities, such as anti-inflammatory, anti-tumor, anti-bacteria, immunoregulatory, anti-hyperuricemia, anti-diuretic and cardioprotection [14,15]. At present, several studies have reported the qualitative and quantitative analysis of the steroidal saponins in DH or DS, and studies regarding the chemical analysis of DH are relatively fewer compared with that of DS [16]. To our best knowledge, there has been little study and discussion on chemical comparison of bioactive steroidal saponins between these two similar herbal medicines from Dioscorea. Moreover, the xanthine oxidase inhibitory activities of DH and DS are still lacking study, and no research has been conducted to investigate relationships between the bioactive compounds and xanthine oxidase inhibitory activities of DH and DS.

The chemical fingerprints can reveal the chemical characteristics of herbal medicines to a certain extent, which could be a convenient and effective tool for evaluation of the uniformity and quality of herbal medicines [17]. The spectrum–effect relationship is a reliable and mature method that can link the chemical fingerprints of herbal medicines with specific bioactivities [18,19]. When combining the results of a pharmacodynamics study and chemical fingerprints data, the spectrum–effect relationship analysis not only can make the chemical composition in fingerprint reflect the corresponding pharmacodynamic effect, but also can clarify the correlation between fingerprint characteristics and pharmacodynamic effect. Thus, the spectrum–effect relationship method has been widely applied in evaluation and screening of the bioactive ingredients from herbal medicines [20].

The present study aimed to compare chemical profiles of DH and DS, and identification of xanthine oxidase inhibitors based on an integrated strategy. Firstly, a high-performance liquid chromatography (HPLC) method was applied to establish the chemical fingerprints of DH and DS samples. Then, chemometric methods including hierarchical clustering analysis (HCA), principal component analysis (PCA) and orthogonal partial least squares discriminant analysis (OPLS-DA) were used to investigate the chemical differences between DH and DS samples. The anti-xanthine oxidase activities of these two herbal medicines were subsequently determined by xanthine oxidase inhibitory assay. The spectrum–effect relationships between the chemical fingerprints and xanthine oxidase inhibitory activities were performed to explore the xanthine oxidase inhibitors by Pearson correlation analysis and partial least squares regression analysis (PLSR). Finally, the xanthine oxidase inhibitors selected from DH and DS were validated, and the possible inhibiting effect on xanthine oxidase was researched by in silico molecular docking.

## 2. Results and Discussion

### 2.1. HPLC Fingerprints Analysis of DH and DS Samples

#### 2.1.1. Optimization of Extraction Condition and HPLC Condition

In order to obtain satisfactory extraction efficiency of DH and DS samples, the extraction conditions, including extraction methods, extraction solvents, extraction time and extraction liquid–solid ratios were optimized. The results showed that both refluxing extraction and ultrasonic extraction had good extraction efficiency. It was found that 70% (*v*/*v*) ethanol was the most efficient extraction solvent among the tested different solvents (100% methanol, 100% ethanol, 70% ethanol and 50% ethanol). In addition, different extraction time (15 min, 30 min and 45 min) and different liquid–solid ratios (10 mL/g, 15 mL/g and 20 mL/g) were optimized, and the optimal extraction efficiency was obtained with the extraction time 15 min and the liquid–solid ratios 15 mL/g. Finally, the extraction condition for DH and DS samples was chosen as ultrasonic extraction with 70% ethanol at liquid–solid ratios 15 mL/g for 30 min.

In order to achieve a rapid and efficient separation of DH and DS samples by HPLC, different mobile phases (water-methanol and water-acetonitrile), different column temperatures (15 °C, 20 °C and 25 °C), and different flow rates (0.80 mL/min, 1.0 mL/min and 1.20 mL/min) were compared and optimized. The result indicated that water-acetonitrile with gradient elution showed the best separation for the analytes. The column temperature was set at 20 °C and the flow rate was selected at 1.00 mL/min in the present study.

#### 2.1.2. Method Validation

The precision, repeatability and stability of the established HPLC method used for chemical fingerprints were validated. As shown in Table 1, the intra-day and inter-day precision (RSDs) of peak areas of nine common peaks were less than 2.67% and 2.49%, respectively. The repeatability and stability presented as RSDs were less than 2.80% and 2.57%, respectively. The results demonstrated that the established HPLC method is suitable for chemical fingerprints analysis of DH and DS samples.

#### 2.1.3. HPLC Fingerprints and Similarity Analysis

**Figure 1:**
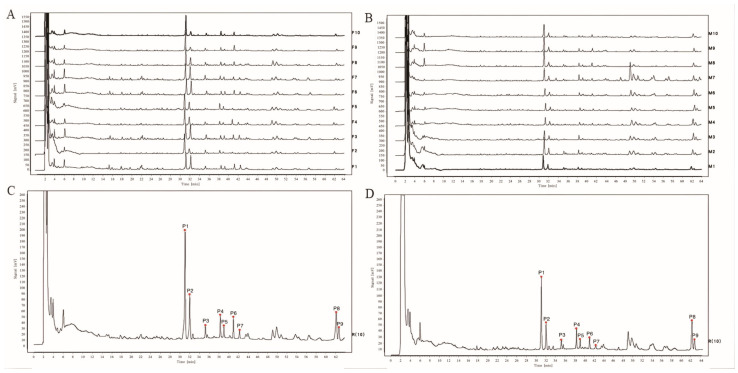
HPLC results of DH and DS samples. (**A**) HPLC fingerprints of 10 batches of DH samples (F1–F10). (**B**) HPLC fingerprints of 10 batches of DS samples (M1–M10). (**C**) The reference fingerprint of DH. (**D**) The reference fingerprint of DS. The peak numbers are consistent with the compound numbers presented in Table 2.

**Figure 2:**
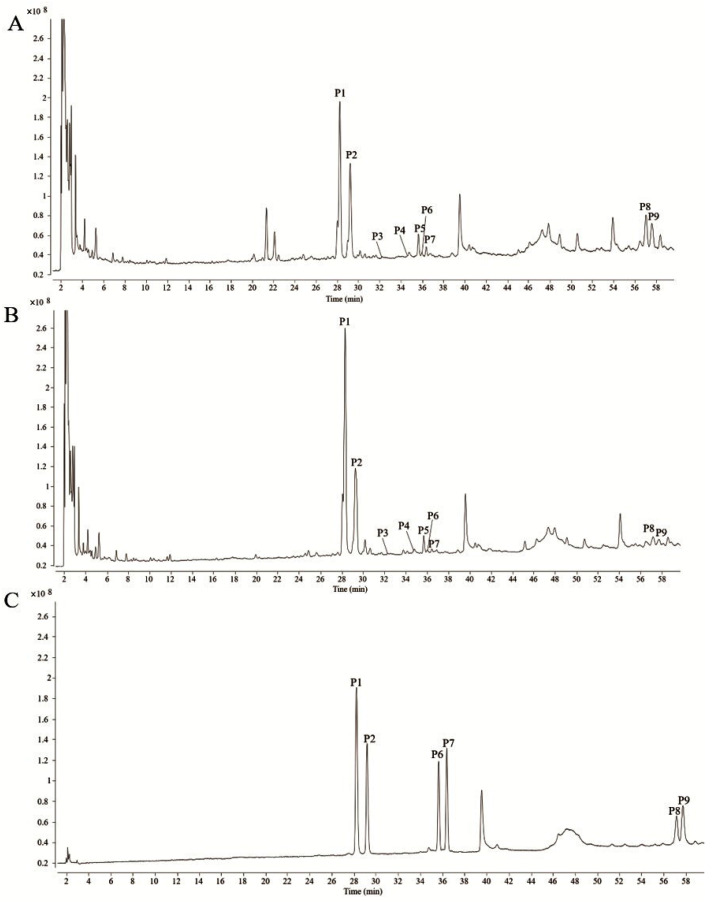
The typical total ion chromatograms of DH (**A**), DS (**B**) and standard solutions (**C**) in positive ion mode by HPLC-Q/TOF-MS. The peak numbers are consistent with the compound numbers presented in Table 2.

**Figure 3:**
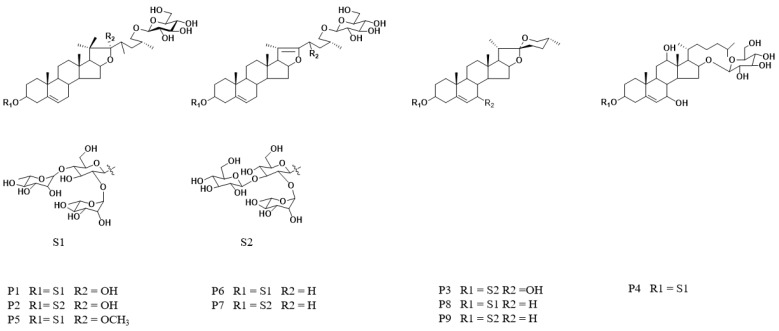
The chemical structures of nine steroidal saponins identified in DH and DS samples. The peak numbers are consistent with the compound numbers presented in Table 2.

Based on the validated HPLC fingerprints method, DH and DS samples were analyzed. The HPLC chromatograms of 10 batches of DH and 10 batches of DS were shown in Figure 1A and 1B, respectively. Then, the chromatograms data of DH and DS samples were saved as CDF format. By the similarity evaluation software (Similarity Evaluation System for Chromatographic Fingerprints of Traditional Chinese Medicines), the HPLC fingerprints and common peaks of DH and DS were automatically matched and selected. The reference fingerprint was generated by median method after multi-point correction and data matching. Finally, a total of nine chromatographic peaks (P1–P9) with good segregation and resolution were recognized as the common peaks in DH and DS samples by similarity evaluation software (Chinese Pharmacopoeia Commission, Beijing, China, version 2012), which represented the similarity among various samples. The reference fingerprints of DH and DS were displayed in Figure 1C and 1D, respectively. The HPLC fingerprints analysis of DH and DS samples showed all the batches of DH and DS samples contained the nine common peaks, which indicated that the chemical profiles of DH and DS were similar. The nine common peaks (P1–P9) in HPLC fingerprints of DH and DS were further identified by HPLC-Q/TOF-MS, and the typical total ion chromatogram of DH and DS samples in positive ion mode is illustrated in Figure 2. Based on fragmentation behaviors, main fragment ions, previous published literature and reference standards, the nine common peaks were identified as protodioscin (P1), protogracillin (P2), (25R)-spirost-5-en-3β,7β-diol-3-O-α-L-arabinofuranosyl(1→4)-[α-L-rhamnopyranosyl-(1→2)]-β-D-glucopyranoside (P3), 3β-O-α-L-rhamnopyranosyl-(1→2)-[α-L-rhamnopyranosyl-(1→4)]-β-D-glucopyranosyl-16β-O-β-D-glucopyranosyl-12β-hydroxycholest-5-ene (P4), methyl protodioscin (P5), pseudoprotodioscin (P6), pseudoprotogtacillin (P7), dioscin (P8) and gracillin (P9), respectively. The HPLC-Q/TOF-MS information such as retention time (RT), chemical formula, ppm errors and main fragment ions is summarized in Table 2, and the chemical structures of the nine steroidal saponins are displayed in Figure 3.

Furthermore, the similarities (correlative coefficient) between the HPLC chromatographic profiles of different batches of DH and DS samples and the reference fingerprints were calculated using the Similarity Evaluation System for Chromatographic Fingerprints of Traditional Chinese Medicines. The correlative coefficients of each DH and DS sample were in the range of 0.923–0.995, which also indicated that these batches of DH and DS samples had similar chemical compositions (Supplementary Table S1).

### 2.2. Chemical Comparison of DH and DS by Chemometric Analysis

The HPLC fingerprints analysis of DH and DS showed that chemical constitutions of DH and DS samples were similar, all the samples have the nine common peaks, but the peak areas of the nine common peaks in different batches of DH and DS samples varied greatly. In order to compare and investigate more information about the chemical difference of DH and DS samples, chemometric analysis including HCA, PCA and OPLS-DA were performed based on the nine common peaks.

#### 2.2.1. Hierarchical Clustering Analysis

**Figure 4:**
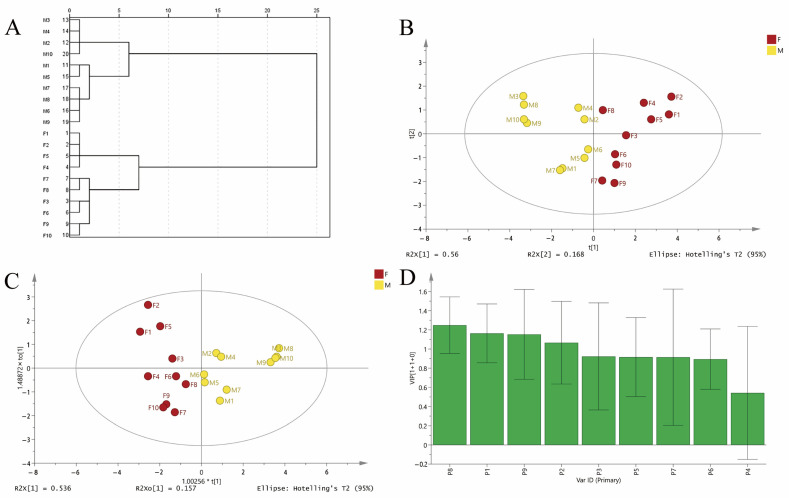
Chemometric analysis of DH and DS samples. DH, F1–F10; DS, M1–M10. (**A**) Dendrogram of HCA of DH and DS samples. (**B**) The score plots of PCA of DH and DS samples. (**C**) The score plots of OPLS-DA of DH and DS samples. (**D**) The VIP values of the nine common peaks.

HCA could divide similar data into different groups by static classification method according to the characteristics of the data [21]. Firstly, HCA was conducted based on the nine common peaks to reveal the relatively homogeneous clusters of DH and DS samples. The HCA dendrogram is displayed in Figure 4A, which showed that the samples could be divided into two categories at Euclidean distance of twenty. DH samples were clustered into one group, and DS samples were clustered into the other group.

#### 2.2.2. Principal Component Analysis and Orthogonal Partial Least Squares Discriminant Analysis

PCA is an unsupervised multivariate pattern recognition method, which could make a holistic evaluation of the samples based on the principle of data dimension reduction. OPLS-DA is a supervised multivariate regression modeling method, which could analyze, classify and reduce the dimensionality of data. These two chemometric methods are widely used in comparison and discrimination of the chemical constitutions of traditional Chinese medicines [22,23]. PCA and OPLS-DA were further applied to effectively display the differences and explore potential chemical markers between DH and DS based on the chromatographic peak areas of the nine common peaks.

In the PCA model, the first two principal components described 72.8% of the variability in the original observations. The score plot of PCA (Figure 4B) showed that 20 batches of samples were divided into two categories corresponding to DH and DS. The result of PCA was similar to that of HCA, which indicated that the chemical difference between DH and DS samples was not a single component contribution, but a multi-component interaction. Thus, OPLS-DA were used to compare the chemical difference and find the potential marker compounds between DH and DS samples. The R^2^X, R^2^Y and Q^2^ parameters of OPLS-DA were 0.693, 0.724 and 0.436, which revealed a good classification and prediction ability. As shown in Figure 4C, the samples were successfully classified into two groups corresponding to DH and DS in OPLS-DA score plot, which was consistent with the PCA result. The variable importance in projection (VIP) values represent the differences of the variables, and variables could be regarded as important roles for the differentiation when the VIP values were more than 1.0. Thus, the VIP values of the nine common peaks in DH and DS samples were calculated (Figure 4D), and the VIP values of P8, P1, P9 and P2 were greater than 1.0. These four common peaks were identified as dioscin (P8), protodioscin (P1), gracillin (P9) and protogracillin (P2). It is worth noting that the peak areas of these four steroidal saponins compounds differed greatly, which indicated that the relative contents of these four steroidal saponins compounds were different in DH and DS samples. The relative contents of protodioscin and protogracillin in DH samples were significantly higher than that of DS samples, while the relative contents of dioscin and gracillin were significantly higher in DS samples compared with DH samples (Supplementary Figure S1). Thus, these four steroidal saponins compounds could be responsible for the significant differences between DH and DS samples, and could be recognized as chemical markers for discrimination and quality evaluation of the two Dioscorea herbal medicines.

### 2.3. Xanthine Oxidase Inhibitory Activities of DH and DS Samples

In the present study, to attempt to study and compare the anti-xanthine oxidase of DH and DS, the DH and DS samples were extracted using 70% (*v*/*v*) ethanol, and the inhibition of xanthine oxidase of the samples were determined by xanthine oxidase inhibition assay. As DH and DS samples were mixtures of multiple constituents, the molar concentrations of the samples were uncertain. Thus, the IC_50_ values of anti-xanthine oxidase activities of DH and DS samples were expressed as mg/mL raw drug equivalents. As shown in Table 3, the DH and DS samples exhibited xanthine oxidase inhibitory activities with the IC_50_ values ranging from 11.89 to 18.93 mg/mL raw drug equivalents. The average IC_50_ values of DH samples (12.98 mg/mL raw drug equivalents) were significantly lower than that of DS samples (17.19 mg/mL raw drug equivalents), which indicated that the anti-xanthine oxidase activities of DH samples were higher than DS samples. The xanthine oxidase inhibition results showed that DH and DS samples exhibited different inhibitory effects on xanthine oxidase, and the common peak areas of DH and DS samples were also different to some extent. The differences of anti-xanthine oxidase activities of DS and DH samples might be due to the presence of various bioactive constituents. Thus, it is necessary to explore the relationships between the bioactive compounds and xanthine oxidase inhibitory activities of DH and DS samples, and find the anti-xanthine oxidase constituents.

### 2.4. Identification of Xanthine Oxidase Inhibitors by Spectrum–Effect Relationship Analysis

#### 2.4.1. Pearson Correlation Analysis

**Figure 5:**
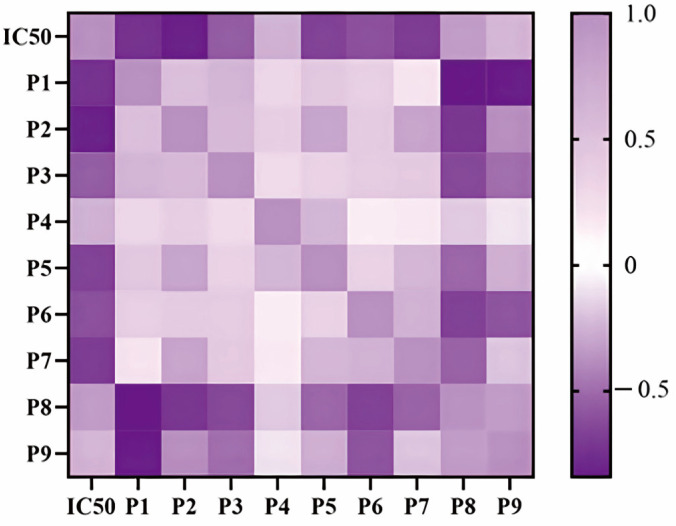
Heatmap analysis of Pearson correlation coefficients of nine common peaks (P1–P9) and anti-xanthine oxidase activities (IC50 values) of different batches of DH and DS samples.

Pearson correlation analysis was firstly applied to study the spectrum–effect relationships between the xanthine oxidase inhibitory activities and bioactive constituents of DH and DS samples. Pearson correlation coefficients between the IC_50_ values of xanthine oxidase inhibitory activities and the nine common peak areas of different samples were calculated. As shown in Figure 5, six common peaks P1, P2, P3, P4, P5 and P7 were negatively correlated to the IC_50_ values, which indicated that these constitutes possessed strong xanthine oxidase inhibitory activities. The Pearson correlation coefficients of P1, P2, P3, P4, P5 and P7 were −0.738, −0.814, −0.564, −0.255, −0.660 and −0.690, respectively. The higher the absolute values of Pearson correlation coefficient were, the greater anti-xanthine oxidase effects the common peaks had.

#### 2.4.2. Partial Least Squares Regression Analysis

**Figure 6:**
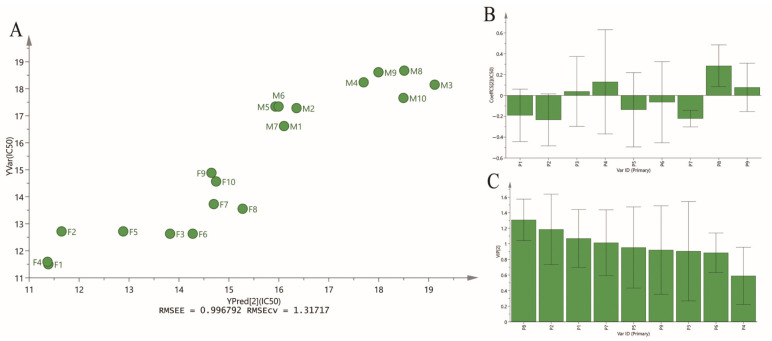
The results of PLSR analysis. (**A**) PLSR linear regression. (**B**) Regression coefficients between nine common peaks and anti-xanthine oxidase activities (IC50 values). (**C**) The VIP values of nine common peaks.

PLSR analysis is a multivariate regression model co-inhering of multivariate data fusion and principal component analysis, and the regression coefficient is considered as the index to exhibit the relative impact of the predictor variables on the response variable for PLSR model [24]. To further investigate the relationships between xanthine oxidase inhibition and bioactive compounds, the PLSR model was established based on the IC_50_ values and common peak areas of DH and DS samples. As shown in Figure 6, five common peaks (P1, P2, P5, P6, P7) showed negative relation to IC_50_ of xanthine oxidase, and the higher the absolute values of regression coefficients were, the stronger xanthine oxidase inhibitory activities the common peaks had. The regression coefficients of P1, P2, P5, P6, P7 were −0.191285, −0.234001, −0.137364, −0.0649832, −0.222602, respectively. Conversely, P3, P4, P8 and P9 showed positive relation to the IC_50_ values, which indicated that these common peaks contributed little to the xanthine oxidase inhibitory activities of DH and DS samples. In PLSR model, the VIP values represent the importance of the variables, and variables with VIP values greater than 1.0 could be considered to be responsible for anti-xanthine oxidase activity. Thus, the VIP values of the nine common peaks were also calculated (Figure 6C). The VIP values of P1, P2, P5, P7, P8 were greater than 1.0.

The Pearson correlation analysis and PLSR analysis showed that some certain components in DH and DS had significant contribution to xanthine oxidase inhibitory activities, and could be recognized as potential anti-xanthine oxidase ingredients. Combined with the results of these two spectrum–effect relationship analyses, four common peaks P1, P2, P5 and P7 identified as protodioscin, protogracillin, methyl protodioscin and pseudoprogracillin were selected as potential xanthine oxidase inhibitors in DH and DS.

#### 2.4.3. Verification of Xanthine Oxidase Inhibitory Activities

**Figure 7:**
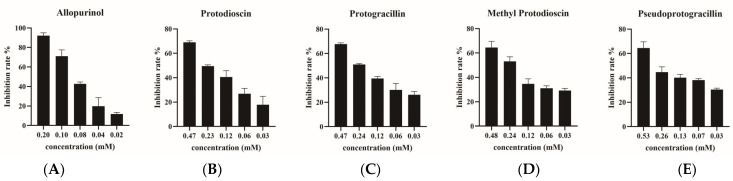
The inhibitory effects of allopurinol (**A**), protodioscin (**B**), protogracillin (**C**), methyl protodioscin (**D**) and pseudoprogracillin (**E**) on xanthine oxidase.

To verify the reliability of above results, the xanthine oxidase inhibitory capacities of the four potential xanthine oxidase inhibitors, protodioscin (P1), protogracillin (P2), methyl protodioscin (P5) and pseudoprogracillin (P7) were determined by xanthine oxidase inhibition assay. A common xanthine oxidase inhibitor allopurinol was used as the positive control. The results showed that allopurinol had a great inhibitory effect with the IC_50_ 0.17 mM, which indicated the established xanthine oxidase inhibition assay was reliable. Then, the xanthine oxidase inhibitory activities of the four steroidal saponins were determined. Each constituent was measured with five concentrations, and the IC_50_ of xanthine oxidase inhibition was calculated. As shown in Figure 7, all the four compounds exhibited dose-dependent inhibitory activities on xanthine oxidase. The IC_50_ values of protodioscin, protogracillin, methyl protodioscin and pseudoprogracillin were 0.20, 0.19, 0.22 and 0.25 mM, which were similar to the IC50 values of positive control (allopurinol). The experimental results indicated that all the four steroidal saponins showed good inhibitory effects on xanthine oxidase, although there was no significant difference among the IC_50_ of these compounds. To sum up, the four compounds might play important roles in the xanthine oxidase activity of DH and DS.

### 2.5. Molecular Docking Experiments

**Figure 8:**
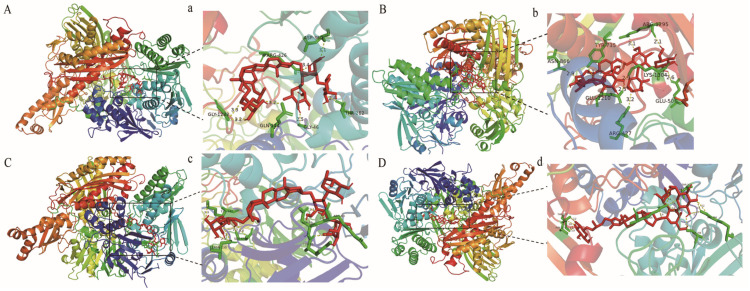
The molecular docking analysis of protodioscin, protogracillin, methyl protodioscin and pseudoprogracillin with xanthine oxidase. (**A**) Preferred docking position of protodioscin on xanthine oxidase. (**a**) Interaction of protodioscin and amino acid residues in xanthine oxidase. (**B**) Preferred docking position of protogracillin on xanthine oxidase. (**b**) Interaction of protogracillin and amino acid residues in xanthine oxidase. (**C**) Preferred docking position of methyl protodioscin on xanthine oxidase. (**c**) Interaction of methyl protodioscin and amino acid residues in xanthine oxidase. (**D**) Preferred docking position of pseudoprogracillin on xanthine oxidase. (**d**) Interaction of pseudoprogracillin and amino acid residues in xanthine oxidase.

To further confirm the xanthine oxidase inhibitory activities of the four selected inhibitors and predict the preferred binding site, molecular docking experiments were performed. The optimal binding affinity of protodioscin, protogracillin, methyl protodioscin and pseudoprogracillin and xanthine oxidase were −10.6, −8.9, −11.1, −11.5 kcal/mol, which indicated that the small molecule compounds and xanthine oxidase had relatively ideal potential activity effects. The interactions between four xanthine oxidase inhibitors and xanthine oxidase are displayed in Figure 8. All the four components were located within the hydrophobic pocket of xanthine oxidase, which is the active site and the coenzyme flavin adenine dinucleotide–binding domain of xanthine oxidase. Protodioscin formed hydrogen bonds with the active-site residues of GLY1233, GLN144, GLY46, THR262, ARG426, ASP360, and protogracillin formed hydrogen bonds with the active-site residues of ASN866, GLU1210, ARG427, GLU560, LYS1304, ARG1295, TRY735. Analogously, methyl protodioscin docked to xanthine oxidase was stabilized by hydrogen bonds to LY340, SER69, ASN130, TYR58, ASP59, ARG60, and pseudoprogracillin docked to xanthine oxidase was stabilized by hydrogen bonds to THR1237, ASN1173, ARG426, SER356, ARG394, THR262. In summary, the main interactions between xanthine oxidase and four xanthine oxidase inhibitors are hydrophobic and hydrogen bonding, which could enable the inhibitors to compete with the substrate for the active site of xanthine oxidase and exhibit the inhibitory activities of xanthine oxidase.

## 3. Materials and Methods

### 3.1. Materials and Reagents

An amount of 10 batches of DH samples and 10 batches of DS samples were purchased from a local Traditional Chinese Medicine market (Anguo, China). The species were authenticated by Associate Professor Long Guo, and the voucher specimens have been deposited in Traditional Chinese Medicine Processing Technology Innovation Center of Hebei Province, Hebei University of Chinese Medicine, Shijiazhuang, China. The origins of DH and DS samples are provided in Supplementary Table S2.

The reference standards of protodioscin, protogracillin, pseudoprotodioscin, pseudoprogracillin, dioscin and gracillin were purchased from Chengdu Push Bio-technology Company (Chengdu, China). The purities of these reference compounds were determined to be higher than 95% by high-performance liquid chromatography with diode array detector. Acetonitrile, methanol and formic acid of HPLC grade were purchased from Merck (Darmstadt, Germany). Ultrapure water was prepared using a milli-Q water purification system from Millipore (Bedford, MA, USA). Other chemicals and reagents were of analytical grade. Xanthine oxidase, xanthine and allopurinol were purchased from Sigma-Aldrich (St. Louis, MO, USA).

### 3.2. Sample Preparation

DH and DS samples were ground into powder and screened through 60 mesh sieves. For HPLC analysis, 1.0 g of each sample was accurately weighed and thoroughly mixed with 70% (*v*/*v*) ethanol (15 mL), then extracted by ultrasonator for 30 min at room temperature. The extracted solution was centrifuged at 13,000 r/min for 10 min and filtered through a 0.22 µm membrane filter prior to injection into the HPLC system.

For xanthine oxidase inhibition assay, 600 μL of supernatant was concentrated in a Termovap Sample Concentrator (Hangzhou Miulab Instruments Co., Ltd., Hangzhou, China) at 30 °C until elimination of solvent. The residue was redissolved in 600 μL PBS.

### 3.3. HPLC Fingerprints

#### 3.3.1. HPLC and HPLC-Q/TOF-MS Conditions

HPLC analysis was performed on the Agilent 1260 HPLC system (Agilent, San Jose, CA, USA) comprising an auto-sampler, a binary pump, a thermostatically controlled column apartment and a diode array detector. Chromatographic separation was conducted on an Agilent ZORBAX Eclipse Plus C18 column (4.6 × 250 mm, 1.8 μm). The mobile phase consists of water (A) and acetonitrile (B) with a gradient elution as follows: 0–15 min, 10%–20%B; 15–30 min, 20%–30%B; 30–35 min, 30%–35%B; 35–45 min, 35%–40%B; 45–60 min, 40%–50%B; 60–65 min, 50%–80%B. The flow rate was maintained at 1.0 mL/min and the column temperature was set at 20 °C. The detection wavelength was set at 203 nm and the injection volume was set at 10 μL.

The HPLC-Q/TOF-MS analysis was performed on an Agilent 1290 UHPLC system coupled with an Agilent 6545 quadrupole time-of-flight mass spectrometer system (Agilent Technologies, Santa Clara, CA, USA). The HPLC chromatographic conditions were the same as HPLC analysis above. The MS acquisition parameters were as follows: drying gas (N_2_) temperature, 320 °C; drying gas (N_2_) flow rate, 10.0 L/min; sheath gas temperature, 350 °C; sheath gas flow (N_2_) rate, 11 L/min; nebulizer gas pressure, 35 psi; fragmentor voltage, 135 V; capillary voltage, 3500 V; collision energy, 40 eV. The analysis was operated in positive mode with the mass range of *m*/*z* 120–1000 Da. Data acquisition was performed with MassHunter Workstation (Agilent Technologies, USA).

#### 3.3.2. Standard Solutions Preparation

Seven reference compounds, including protodioscin, protogracillin, methyl protodioscin, pseudoprotodioscin, pseudoprotogracillin, dioscin and gracillin were weighed accurately and dissolved in 70% (*v*/*v*) ethanol to prepare the stock standard solutions with the concentrations of 4.5 mg/mL. Then, the working standard solutions were prepared by diluting the stock standard solution with 70% (*v*/*v*) ethanol to a series of proper concentrations. All the solutions were stored at 4 °C before analysis.

#### 3.3.3. Method Validation

To ensure the reliability of the established HPLC method, the precision, repeatability and stability were validated. Sample solutions were prepared according to Section 2.2. The precision was determined by the intra- and inter-day variations. For intra-day precision, the same sample was injected and analyzed for 6 consecutive times within the same day, while for inter-day precision, the sample was examined in duplicates for consecutive three days. For the repeatability test, six samples were extracted and analyzed independently. To confirm the stability, the same samples were stored at room temperature and analyzed at 0, 2, 6, 8, 12 and 24 h.

#### 3.3.4. HPLC Fingerprints Analysis

An amount of 10 batches of DH samples and 10 batches of DS samples were analyzed by the established HPLC method to obtain the chromatograms and the chromatograms data were saved as CDF format. The HPLC fingerprints of DH and DS were automatically matched using a similarity evaluation software, Similarity Evaluation System for Chromatographic Fingerprints of Traditional Chinese Medicines, respectively (Chinese Pharmacopoeia Commission, Beijing, China. version 2012). The reference fingerprint was generated by median method after multi-point correction and data matching. The similarities between the reference fingerprint and the chromatograms of the samples were also calculated by the software.

### 3.4. Xanthine Oxidase Inhibitory Assay

The xanthine oxidase inhibitory activities of DH and DS samples were determined by xanthine oxidase inhibitory activity assay in vitro [25], which was based on the increase in absorbance at 295 nm due to the production of uric acid from xanthine. In brief, 50 µL of DH or DS samples, 30 µL of phosphate buffer (70 Mm, pH 7.5), and 40 µL of xanthine oxidase (0.05 U/mL) were added into a 96-well plate. After preincubation at 25 °C for 8 min, 60 µL of xanthine (300 µM) was added. After incubation at 25 °C for 15 min, the reaction was stopped by adding 20 µL of HCl (1.0 M). The absorbance of the mixture solution was recorded at 295 nm by a microplate reader. Allopurinol was used as a positive control.

The control sample was prepared by adding PBS instead of tested sample. The background sample was prepared by replacing tested sample with the same volume of PBS. The blank sample was prepared by adding PBS instead of xanthine oxidase solution. All experiments were repeated three times. The inhibition ratio of xanthine oxidase was calculated as follows:

Inhibition ratio (%) = [1 − (test sample-background sample/control sample-blank sample)] × 100

DH and DS samples were prepared in a series of concentrations. The xanthine oxidase inhibitory activities of DH and DS samples were evaluated by IC_50_ values (the concentration of the sample inhibited 50% the activity of the xanthine oxidase, and the IC_50_ values were calculated by a logarithmic regression curve. As DH and DS samples were mixtures of multiple constituents, the molar concentrations were uncertain. Thus, the IC_50_ values of anti-xanthine oxidase activities of DH and DS samples were expressed as mg/mL raw drug equivalents.

### 3.5. Chemometric Analysis

For HCA, the peak areas of the common peaks of DH and DS were imported into SPSS 22.0 software (SPSS Inc., Chicago, IL, USA). After standardized processing, the cosine distance between samples was calculated and the inter-group connection method was performed. The PCA and OPLS-DA were established based on the peak areas of the common peaks of DH and DS samples using SIMCA 14.0 software (Umetrics, Umea, Sweden).

### 3.6. Spectrum–Effect Relationship Analysis

#### 3.6.1. Pearson Correlation Analysis

Pearson correlation analysis is a multivariate statistical model, which is applied to extract factors that have the greatest impact on the outcome variables and maximize the relationships between the two sets of variables. Taking the Pearson correlation coefficient as an index, the peak areas of the common peaks of DH and DS samples in the HPLC fingerprints were recognized as one set of variables, and the xanthine oxidase inhibition ratios (IC_50_ values) as the other set. Pearson correlation coefficients between common peaks and IC_50_ values were analyzed by SPSS 22.0 statistics software (SPSS Inc., Chicago, IL, USA).

#### 3.6.2. Partial Least Squares Regression Analysis

PLSR is a multivariate regression model combination of multiple linear regression, canonical correlation analysis and principal component analysis [26], which has the advantages of low computation and high prediction accuracy. In this study, PLSR was also used to model the correlation between the common peaks and xanthine oxidase inhibitory activities of DH and DS samples. The peak areas of common peak areas were used as the independent X variables, and the xanthine oxidase inhibitory activities (IC_50_ values) were used as dependent Y variables. The relative impact of the predictor variables on the response variable was reflected through their regression coefficient. The PLSR was performed on Simca-P 14.0 software (Umetrics, Umea, Sweden).

### 3.7. Molecular Docking Experiments

To explore the possible binding of inhibitors with xanthine oxidase, molecular docking experiments were performed using AutoDock Vina. The initial structure of xanthine oxidase (PDB ID: 1FIQ) was obtained from RCSB protein data bank (http://www.rcsb.org/). The 3D structure was downloaded from Pubchem (https://pubchem.ncbi.nlm.nih.gov/). Unnecessary water molecules were removed and polar hydrogen atoms were added, and each atom of the protein was assigned Kollman charges. The AutoDock Vina program was used to calculate the possible conformation of structure that binds to the xanthine oxidase as well as the binding affinity, and PYMOL was further applied to investigate the probable binding interactions between inhibitors and xanthine oxidase.

## 4. Conclusions

In this present work, the chemical fingerprints of DH and DS were established. HCA, PCA and OPLS-DA were performed to compare and discriminate DH and DS based on the fingerprints data, and protodioscin, protogracillin, dioscin, gracillin were selected as chemical markers for the differences. Then, the spectrum–effect relationship between fingerprints and xanthine oxidase inhibitory activities of DH and DS were established by Pearson correlation analysis and PLSR analysis. Four steroidal saponins, including protodioscin, protogracillin, methyl protodioscin and pseudoprogracillin might be the potential xanthine oxidase inhibitors in DH and DS. The xanthine oxidase inhibitory activities of the four selected steroidal saponins were validated by xanthine oxidase inhibition assay and molecular docking experiments. This present study might facilitate better understanding of the chemical difference of DH and DS, and provide evidence for the anti-xanthine oxidase activities and identify the xanthine oxidase inhibitors of DH and DS.

## Figures and Tables

**Table 1:** Precision, repeatability and stability of HPLC fingerprints method.

Common Peaks	Intra-Day Precision (RSD%)	Inter-Day Precision (RSD%)	Repeatability (RSD%)	Stability (RSD%)
P1	0.66	0.41	1.31	2.33
P2	0.71	0.50	1.23	1.70
P3	0.83	0.80	2.03	2.57
P4	2.67	0.81	1.68	0.79
P5	1.24	0.72	1.98	1.40
P6	1.87	2.49	2.80	2.34
P7	0.56	1.44	2.30	1.98
P8	1.40	0.63	0.69	2.46
P9	2.24	1.73	2.63	2.42

**Table 2:** The HPLC-Q/TOF-MS information of the nine steroidal saponins identified in DH and DS samples.

NO.	RT	Compound	Chemical Formula	Theoretical Mass (*m*/*z*)	Error (ppm)	Measured Mass (*m*/*z*)	Fragment Ions (*m*/*z*)
P1	28.18	Protodioscin *	C_51_H_84_O_22_	1048.5449	2.87	1031.5450 [M+H−H_2_O]^+^	1031.5426, 869.49302, 725.3763, 579.3129, 415.3203, 379.2054, 253.1950, 129.0552, 85.0827
P2	29.183	Protogracillin *	C_57_H_84_O_23_	1064.5398	2.94	1047.5401 [M+H−H_2_O]^+^	1047.5350, 885.4866, 723.4337, 579.3179, 415.3211, 379.3009, 253.1957, 129.0549, 85.0284
P3	32.301	(25R)-spirost-5-en-3β,7β-diol-3-O-α-L-arabinofuranosyl(1→4)-[α-L-rhamnopyranosyl-(1→2)]-β-D-glucopyranoside	C_45_H_72_O_18_	900.4698	−2.32	923.4611 [M+Na]^+^	901.4785, 739.4253, 593.3669, 346.2947
P4	34.552	3β-O-α-L-rhamnopyranosyl-(1→2)- [α-L-rhamnopyranosyl-(1→4)]-β-D-glucopyranosyl-16β-O-β-D-glucopyranosyl-12β-hydroxycholest-5-ene	C_51_H_86_O_21_	1034.5647	−1.43	1057.5534 [M+Na]^+^	1035.5611, 889.6539, 743.5023, 579.1387
P5	35.636	Methyl Protodioscin	C_52_H_86_O_22_	1062.5605	2.37	1031.5449 [M+H−CH_3_OH]^+^	1031.5445, 869.4905, 725.3745, 577.3735, 415.3212, 253.1234, 129.0547, 85.0286
P6	35.986	Pseudoprotodioscin *	C_51_H_82_O_21_	1030.5343	2.82	1031.478 [M+H]^+^	1031.5445, 869.4901, 725.3741, 577.3740, 415.3211, 379.2926, 253.1953, 147.0652, 129.0548, 85.0287
P7	36.368	Pseudoprotogtacillin *	C_51_H_82_O_22_	1046.5292	2.27	1047.5392 [M+H]^+^	1047.5379, 885.4804, 723.4314, 577.3744, 415.5212, 397.3085, 309.1188, 147.0656, 129.0538
P8	57.060	Dioscin *	C_45_H_72_O_16_	868.4815	2.92	869.4916 [M+H]^+^	869.4917, 723.4308, 577.3734, 415.3218, 293.1428, 253.1954, 129.0545
P9	57.601	Gracillin *	C_45_H_72_O_17_	884.4764	1.9	885.4855 [M+H]^+^	885.4845, 723.4328, 577.3854, 415.3214, 397.3103, 253.1954, 85.0285

* Identified by comparing with reference compounds.

**Table 3:** Inhibitory effects (IC_50_ values) of different batches of DH (F1–F10) and DS (M1–M10) samples on xanthine oxidase. (mg/mL raw drug equivalents.)

NO.	IC_50_ (mg/mL)	NO.	IC_50_ (mg/mL)
F1	11.72 ± 0.21	M1	16.54 ± 0.2
F2	12.63 ± 0.11	M2	17.25 ± 0.04
F3	12.41 ± 0.18	M3	18.03 ± 0.12
F4	11.55 ± 0.10	M4	18.18 ± 0.26
F5	12.52 ± 0.22	M5	17.32 ± 0.20
F6	12.53 ± 0.27	M6	17.57 ± 0.26
F7	13.67 ± 0.07	M7	16.52 ± 0.17
F8	13.37 ± 0.16	M8	18.73 ± 0.18
F9	14.83 ± 0.12	M9	18.33 ± 0.23
F10	14.58 ± 0.12	M10	17.61 ± 0.38

## Data Availability

Data are included within the manuscript or the Supplementary Materials.

## Supplementary Materials

The following supporting information can be downloaded at https://www.mdpi.com/article/10.3390/molecules28248116/s1, Figure S1: The average peak areas of protodioscin (A), protogracillin (B), dioscin (C) and gracillin (D) in DH and DS samples (** *p* < 0.01, *** *p* < 0.001); Table S1: Similarities of 10 batches of DH samples (F1–F10) and 10 batches of DS samples (M1–M10); Table S2: Samples of Dioscoreae hypoglaucae Rhizoma (DH) and Dioscoreae spongiosae Rhizoma (DS).
