# Supplementary material for: Chemical Comparison and Identification of Xanthine Oxidase Inhibitors of Dioscoreae Hypoglaucae Rhizoma and Dioscoreae Spongiosae Rhizoma by Chemometric Analysis and Spectrum–Effect Relationship
Source: Molecules. 2023 Dec 15;28(24):8116. doi: 10.3390/molecules28248116 (PMC10745721; doi:10.3390/molecules28248116)
Supplement: Supplementary file 1 [file molecules-28-08116-s001.zip › molecules-2762811-supplementary.pdf]

Supplementary Materials

Figure S1 The average peak areas of protodioscin (A), protogracillin (B), dioscin (C) and gracillin (D) in DH and DS samples (** $P < 0.01$, *** $P < 0.001$).

Table S1 Similarities of 10 batches of DH samples (F1-F10) and 10 batches of DS samples (M1-M10).

Table S2 Samples of Dioscoreae hypoglaucae Rhizoma (DH) and Dioscoreae spongiosae Rhizoma (DS).

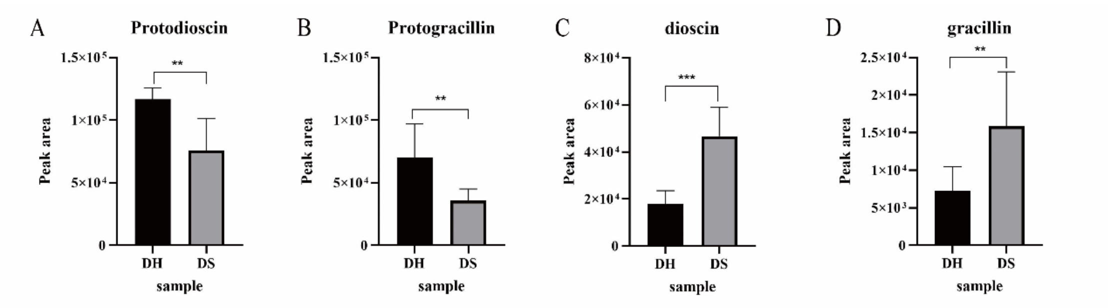

Figure S1 The average peak areas of protodioscin (A), protogracillin (B), dioscin (C) and gracillin (D) in DH and DS samples (** $P < 0.01$, *** $P < 0.001$).

Table S1 Similarities of 10 batches of DH samples (F1-F10) and 10 batches of DS samples (M1-M10).

NO.	Similarity	NO.	Similarity
F1	0.980	M1	0.967
F2	0.971	M2	0.983
F3	0.991	M3	0.938
F4	0.982	M4	0.952
F5	0.982	M5	0.944
F6	0.98	M6	0.988
F7	0.994	M7	0.976
F8	0.974	M8	0.98
F9	0.976	M9	0.986
F10	0.986	M10	0.982

Table S2 Samples of Dioscoreae hypoglaucae Rhizoma (DH) and Dioscoreae spongiosae Rhizoma (DS).

NO.	Sample	Origins	NO.	Sample	Origins
F1	DH	Hunan, China	M1	DS	Jiangxi, China
F2	DH	Hunan, China	M2	DS	Jiangxi, China
F3	DH	Hunan, China	M3	DS	Jiangxi, China
F4	DH	Hunan, China	M4	DS	Zhejiang, China
F5	DH	Hunan, China	M5	DS	Zhejiang, China
F6	DH	Fujian, China	M6	DS	Zhejiang, China
F7	DH	Fujian, China	M7	DS	Zhejiang, China
F8	DH	Fujian, China	M8	DS	Sichuan, China
F9	DH	Zhejiang, China	M9	DS	Sichuan, China
F10	DH	Zhejiang, China	M10	DS	Sichuan, China
